# The PKR/P38/RIPK1 Signaling Pathway as a Therapeutic Target in Alzheimer’s Disease

**DOI:** 10.3390/ijms22063136

**Published:** 2021-03-19

**Authors:** Jacques Hugon, Claire Paquet

**Affiliations:** Center of Cognitive Neurology, Lariboisiere FW Hospital, University of Paris, AP-HP, 75010 Paris, France; claire.paquet@inserm.fr

**Keywords:** Alzheimer, kinases, signaling pathways, neuroinflammation, combination therapy

## Abstract

Neuropathological lesions in Alzheimer’s disease (AD) include amyloid plaques formed by the accumulation of amyloid peptides, neurofibrillary tangles made of hyperphosphorylated tau protein, synaptic and neuronal degenerations, and neuroinflammation. The cause of AD is unknown, but according to the amyloid hypothesis, amyloid oligomers could lead to the activation of kinases such as eukaryotic translation initiation factor 2-alpha kinase 2 (PKR), p38, and receptor-interacting serine/threonine-protein kinase 1 (RIPK1), which all belong to the same stress-activated pathway. Many toxic kinase activations have been described in AD patients and in experimental models. A p38 mitogen-activated protein kinase inhibitor was recently tested in clinical trials but with unsuccessful results. The complex PKR/P38/RIPK1 (PKR/dual specificity mitogen-activated protein kinase kinase 6 (MKK6)/P38/MAP kinase-activated protein kinase 2 (MK2)/RIPK1) is highly activated in AD brains and in the brains of AD transgenic animals. To delineate the implication of this pathway in AD, we carried out a search on PubMed including PKR/MKK6/p38/MK2/RIPK1, Alzheimer, and therapeutics. The involvement of this signaling pathway in the genesis of AD lesions, including Aβ accumulations and tau phosphorylation as well as cognitive decline, is demonstrated by the reports described in this review. A future combination strategy with kinase inhibitors should be envisaged to modulate the consequences for neurons and other brain cells linked to the abnormal activation of this pathway.

## 1. Introduction

Alzheimer’s disease (AD) is clinically marked by memory disturbances followed by aphasia, apraxia, and agnosia and is later associated with behavioral symptoms [1]. Neuropathological lesions include extracellular amyloid plaques made of aggregated amyloid-beta peptides including Aβ1-42 and neurofibrillary tangles (NFTs) formed by hyperphosphorylated tau protein, neuroinflammation, and synaptic and neuronal loss [2]. The causes of AD are unknown. According to the amyloid cascade hypothesis [3], Aβ1-42 monomers and oligomers could be responsible for neurotoxic consequences associated to activation of kinases, tau hyperphosphorylation, microglial activation, and inflammation, leading to neuronal death. Other hypotheses of the origin of AD lesions have been proposed and include early tau accumulation, neuroinflammation, lipid metabolism, innate immunity, and endocytosis [4]. Recent advances in cerebrospinal fluid (CSF), blood, and imaging biomarker research have demonstrated that Aβ1-42 accumulation starts decades before the first clinical sign, marked by reduced CSF levels, rapidly followed by CSF tau anomalies, and then CSF triggering receptor expressed on myeloid cells 2 (TREM2) and neurogranin disturbances [5]. Based on these different features, new biological and imaging biomarkers are being detected to make a high-confidence diagnosis [6]. Metabolic and nutritional profiles are relevant contributors to the disease’s development and progression [7]. So far, disease-modifying treatments targeting Aβ, tau, or other proteins have not shown positive effects in AD clinical trials [8,9,10,11,12]. According to the amyloid cascade hypothesis [3], the presence of abnormal kinase activations and neuroinflammation linked to amyloid accumulation in AD brain lesions has been largely documented and leads to tau phosphorylation, Aβ production, glutamate toxicity, and neuronal degeneration [13,14,15,16]. The discovery of new drugs modulating abnormal signaling pathways involved in the pathophysiology of AD is urgently needed. The goal of the present review is to analyze the involvement of a specific signaling pathway in AD, starting with the cellular triggering of the eukaryotic initiation factor 2 α (eIF2α) kinase II (PKR) in brain cells and leading to the final activation of the receptor-interacting serine/threonine-protein kinase 1 (RIPK1). All involved kinases were shown to be activated in AD brains or AD CSF, and the approach of inhibitory combination therapy could be an interesting new way to attenuate the detrimental consequences of their activations in AD brains. The possible targets could include Aβ production, tau hyperphosphorylation, triggering of inflammatory pathways, and synaptic and neuronal degenerations. We carried out a search on PubMed including the terms PKR or MKK6, p38 or MK2 or RIPK1 and Alzheimer’s and therapeutics, and we selected the relevant articles associating these kinases and the articles showing the implication of these kinases in AD. The literature was searched on PubMed including the words PKR, MKK6, p38, MK2, RIPK1, and Alzheimer’s disease.

## 2. The PKR/p38/RIPK1 Pathway

### The Physiological Pathway

**Figure 1:**
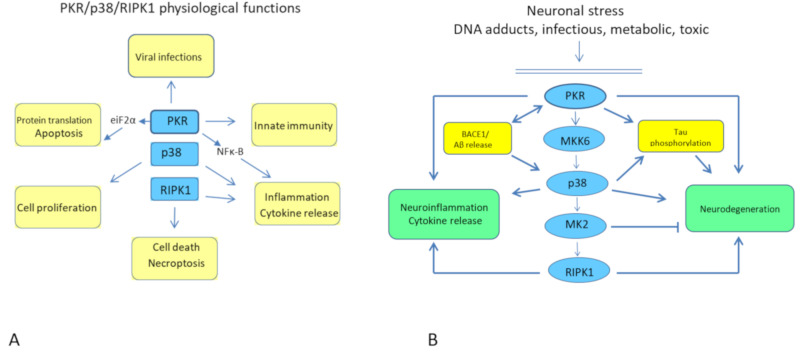
(**A**) Physiological functions of the various kinases included *in the PKR/p38/RIPK1 pathway*. PKR controls the initiation of protein translation, the partial replication of viruses during infections, and is involved in the modulation of inflammatory signals associated with innate immunity and apoptosis. p38 is implicated in cell proliferation and cell death as well as inflammatory pathways. RIPK1 controls necroptosis (associated with RIPK3) and modulates cytokine release and inflammation. (**B**) Pathological consequences of the activation of the PKR/p38/RIPK1 pathway during neuronal stress and Alzheimer’s disease (AD). The abnormal activation of this pathway in the brain is linked to Aβ oligomers’ accumulation in AD brains and can lead to increased levels of beta-site APP cleaving enzyme 1BACE1, inducing more Aβ synthesis, and to enhanced synthesis of the tau protein and its abnormal phosphorylation. In addition, neuroinflammatory, apoptosis, and necroptosis signals are highly exacerbated upon activation of the PKR/p38/RIPK1 pathological signaling pathway in AD. Note: Eukaryotic translation initiation factor 2-alpha kinase 2 (PKR); receptor-interacting serine/threonine-protein kinase 1 (RIPK1); receptor-interacting serine/threonine-protein kinase 3 (RIPK3); dual specificity mitogen-activated protein kinase kinase 6 (MKK6); MAP kinase-activated protein kinase 2 (MK2).

Most of the kinases of this pathway are ubiquitously expressed in human cells, including immune cells such as macrophages, lymphocytes, and microglial cells. This pathway is depicted in Figure 1A. PKR is implicated in many physiological functions including viral protection, control of translation initiation, apoptosis and cell proliferation, innate immunity, and inflammation [17]. PKR autophosphorylation and activation reverse translation and protein synthesis via eIF2α phosphorylation, inducing apoptosis in response to many cell stresses. PKR is part of the Integrated Stress Response (ISR) together with PKR-like endoplasmic reticulum kinase (PERK), eukaryotic translation initiation factor 2-alpha kinase 1 (HRI), and eukaryotic translation initiation factor 2-alpha kinase 4 (GCN2) [18]. All these kinases phosphorylate eIF2α, blocking translation initiation. PKR also has the possibility to activate the nuclear factor κ-B pathway, promoting inflammation and cytokine release. PKR is a ubiquitously expressed serine–threonine protein kinase and is implicated in the innate immune viral response induced by interferon. In addition, activated PKR is associated with inflammation and immune control through the action of several stimuli such as inflammatory cytokine release, bacterial infection, DNA damage, and damage-associated molecular patterns (DAMPs) [13]. The p38 kinase is mainly involved in cell proliferation, apoptosis, and inflammation [19]. There are four p38 kinases in the family (α, β, γ, and δ) encoded by different genes and with various patterns of tissue expression. The kinase RIPK1 is implicated in the control of necroptosis with RIPK3, cell apoptosis, cytokine release, and inflammation [20]. Necroptosis is a type of controlled necrosis triggered by RIPK1 and/or RIPK3 when caspases are inactivated.

**Figure 2:**
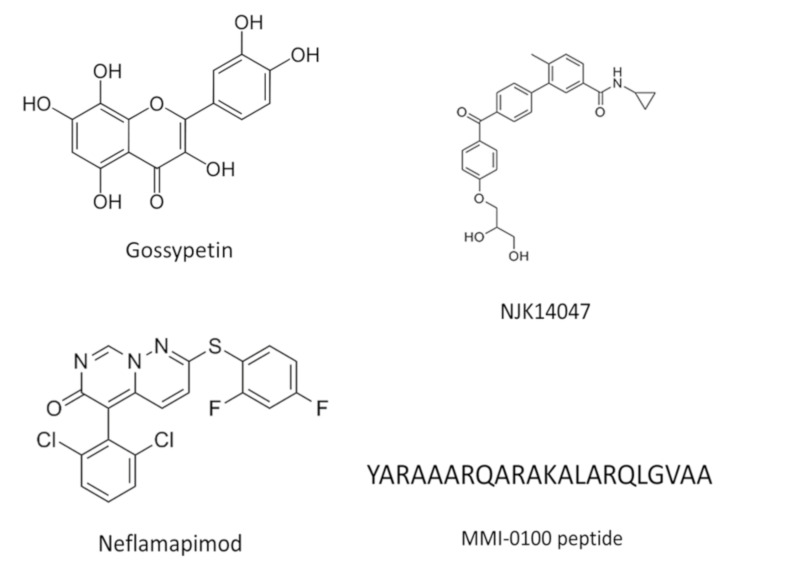
Chemical structures of several inhibitors described in the text.

The abnormal cellular consequences of this pathway’s activation are shown in Figure 1B. Most of the kinases involved in this signaling pathway were already implicated in AD pathogenesis. For example, Aβ1-42 is able to trigger the autophosphorylation of PKR in neuronal cultures in vitro, leading to neuronal apoptosis that is attenuated in neurons from PKR knockout mice [21]. This molecular event is calcium-dependent and is also controlled by the activation of caspase 3 [22]. PKR is known to induce the activation of dual specificity mitogen-activated protein kinase kinase 6 (MKK6), which, in turn, triggers p38 kinase [23,24]. Finally, recent reports have highlighted the control of RIPK1 signaling by the p38/MK2 complex [25,26,27]. We will now describe the possible involvement of each kinase of this pathway in AD pathogenesis and the attempts to use some kinase inhibitors in AD models or in clinical trials. Previous results are summarized in Table 1 and the chemical structures of several inhibitors are shown in Figure 2.

## 3. PKR and AD

PKR is a stress and pro-apoptotic kinase activated by viruses, interferons, tumor necrosis factor α (TNFα), endoplasmic reticulum (ER) stress, reactive oxygen species (ROS), and calcium. PKR is activated by autophosphorylation on Ser446 and Ser451 [16]. PKR is a regulator of cellular response to DNA adducts [28]. In addition, PKR is activated during the Integrated Stress Response (ISR), which is implicated in memory control and in many human pathological conditions [18]. PKR-like endoplasmic reticulum kinase (PERK) is also activated during ISR triggering as well as during the Unfolded Protein Response (UPS). Both stress kinases trigger the phosphorylation of eIF2α, leading to protein initiation blockade. We have previously shown that aggregated Aβ can induce PKR activation [21]. Levels of activated PKR are enhanced in the brains and CSF of AD patients [21,29,30], and CSF levels of phosphorylated PKR are associated with cognitive decline [31]. Increased levels of brain PKR were also revealed in Parkinson’s disease and Huntington’s disease [16]. PKR controls BACE1 levels through eIF2α phosphorylation [32]. In 5XFAD mouse models (expressing human APP and PSEN1 transgenes associating five AD-linked mutations: the Swedish (K670N/M671L), Florida (I716V), and London (V717I) mutations in APP, and the M146L and L286V mutations in PSEN1, the genetic blockade of PKR reduced cognitive memory impairment, neurodegeneration, neuroinflammation, and brain Aβ 1-42 accumulation [33]. PKR activation can induce tau phosphorylation and control tau protein synthesis [34,35]. PKR is also implicated in inflammasome and inflammatory signals controlling, at various molecular levels, cytokine release in immune cells [13]. An earlier study has shown that PKR triggered by doucble-stranded RNA activates dual specificity mitogen-activated protein kinase kinase 6 (MKK6) in vitro and regulates p38 mitogen-activated protein kinase (MAPK) induction, initiating a signaling cascade able to contribute to cell stress and inflammation [23]. Pharmacological PKR inhibition can reach the brain [36] and experimentally reduced brain Aβ accumulation in a thiamine-deficient model in mice [37]. However, so far, no PKR inhibitor has been tested in humans due to unavailability of compounds. Probably, most of the experimental PKR inhibitors used in animal studies revealed organ toxicities, preventing them from being tested in phase 1 human studies [36,37]. PKR accumulation was also described in the brain of patients with Huntington’s disease [38]. The discovery of PKR inhibitors is urgently needed to test their actions in various neurological conditions marked by ISR activation.

## 4. MKK6 and AD

The kinase MKK6 is one of the upstream activators of p38 MAPK, and a previous report has demonstrated that MKK6 can be physically associated with tau protein and can participate in abnormal tau phosphorylation at epitopes such as Ser202/Thr205 and Ser204 through p38 MAPK activation [39]. In addition, a neuropathological study involving 21 AD patients and 18 controls revealed that MKK6 staining was increased in AD brains and specifically associated with granulovacuolar structures in susceptible neurons of the hippocampus and the cortex [40]. The labeling of MKK6 overlapped with p38 MAPK staining, suggesting a functional and mechanistic link. Immunoblots showed increased levels of MKK6 protein in AD brains as compared to control brains. These granulovacuolar neuronal deposits were also described in similar regions of AD brains for phosphorylated PKR stainings [41]. Brain Lewy bodies, made of α-synuclein accumulations, are the characteristic lesions of Parkinson’s disease and are found in one-third of AD patients [42]. Experimental genetic downregulation of MKK6 or p38 MAPK kinases enhances proteasome activity, which, in turn, decreases the cellular levels of both overexpressed and endogenous α-synuclein [43]. The induction of this proteasome pathway could also contribute to reducing Aβ1-42 accumulation, but to our knowledge, no MKK6 inhibitor has been tested in clinical trials including AD patients. Surprisingly, in cancer research, the flavonoid gossypetin was shown to inhibit MKK3 and MKK6 kinases but was also able to block the cell cycle and induce apoptosis in cancer cells by activating caspase 8 and caspase 3 [44]. Further studies will be needed to explore the usefulness of MKK6 inhibitors in AD to determine the fate of neurons exposed to these inhibitors.

## 5. P38 and AD

Four spliced variants of p38 MAPK (MAPK11) have been described: p38 α, p38 β, p38 γ, and p38 δ. Only P38 α and p38 β are ubiquitously expressed in human tissues [45]. One of the first reports showing that the stress-activated p38 kinase was activated in AD brains was published in 1998 [46]. p38 MAPK is implicated in many pathological processes in the pathogenesis of AD [47]. Numerous studies have revealed that p38 MAPK controls cytokine production by glial cells [48], induces tau phosphorylation and oxidative stress [49], and can be responsible for synaptic dysfunction and cognitive deficits [50]. p38 MAPK is activated early in AD brains and is not always linked to neurofibrillary tangles (NFTs) [51]. As we have seen previously, p38 is activated by PKR through MKK6 activation [24]. This pathway is triggered during the activation of innate immunity by lipopolysaccharide (LPS) in cell cultures, and the production of interleukin-6 is deficient in PKR knockout cells following LPS exposure [52]. The use of p38 inhibitors in AD transgenic mice was extensively studied [53,54,55,56,57], and, for example, a report using NJK14047 has shown interesting results including a reduction in brain Aβ accumulation, attenuated spatial learning deficit, and decreased neurotoxicity and cerebral neuroinflammatory markers [56]. These experimental results paved the way for testing p38 inhibitors in AD patients. In a recent report, 16 patients with early AD received neflamapimod, a selective p38 α inhibitor, at 40 or 125 mg per day for 12 weeks [58,59]. The results showed an improvement from baseline in mean Wechsler Memory Scales immediate and delayed recalls on day 28 and on day 84. Unfortunately, a double-blinded placebo-controlled study including 161 people with biomarker-confirmed AD (called REVERSE-SD) treated with 40 mg twice daily for 24 weeks failed to meet its primary endpoint in improving episodic memory [59]. CSF tau and phosphorylated tau were significantly reduced in the treated groups. Another study using neflamapimod in 91 patients with Lewy body disease has shown that neflamapimod can modulate cognitive decline in these patients [60].

## 6. MK2 and AD

MAPK-activated protein kinase 2 (MK2), a kinase downstream of p38, is involved in the metabolism of TNFα [26]. There is a canonical role of the complex p38/MK2, in which cell stress (for example, LPS exposure) leads to the induction of the p38/MK2 couple and the initiation of TNFα synthesis and inflammation. TNFα production is regulated by the MK2 substrate tristetrapolin. In the non-canonical role, MK2 induces another substrate, RIPK1, and modulates signals linked to TNFα receptor 1activation. In 2006, a report demonstrated that MK2 expression was increased in the brains of AD transgenic mice and MK2 levels correlated with Aβ deposition, microglial activation, and TNFα levels [61]. In addition, cortical neurons co-cultured with LPS + interferon-γ (IFN-γ) or Aβ 1-42-stimulated MK2−/− microglial cells were protected from microglial cell-induced neuronal toxicity as compared to co-cultures using MK2+/+ microglial cells. The authors proposed that MK2 could have a role in AD brain pathology. Because many p38α inhibitors were not successful in clinical development due to their toxicological profiles inducing apoptosis [62,63,64], recent experimental attempts were initiated to find new MK2 inhibitors that could serve as anti-inflammatory agents [65]. For example, an in vitro study revealed that MK2 inhibitors could be superior to p38 inhibitors because these latter inhibitors induce increased phosphorylated C-Jun kinase (pJNK) and caspase 3 activations (leading to possible increased cellular apoptosis) as compared to MK2 inhibitors [66]. A recent report has demonstrated that the new compound CDD-450 selectively blocks the kinase MK2 while sparing p38 α activation and activating transcription factor 2 (ATF2) [67]. CDD-450 could decrease, in animals exposed to LPS endotoxemia, IL-1β and TNFα production while sparing tachyphylaxis associated with global p38 α inhibition. MK2 inhibition was recently explored in an AD experimental model using a new anti-inflammatory chemical, MMI-0100, which is a cell-penetrating peptide inhibitor of MK2 [68]. After intra-cerebroventricular (ICV) or hippocampal injections of Aβ or LPS, ICV or intranasal administrations of MMI-0100 ameliorated memory tasks explored by object recognition, suppressed LPS-induced activation of microglia and astrocytes, and clearly reduced the release of inflammatory cytokines via selective MK2 inhibition without modulating p38 α or JNK. Previous works have revealed that selective MK2 inhibition modulates Aβ or LPS toxicities, but recent studies have demonstrated that MK2 can also control the kinase RIPK1, which is possibly involved in AD pathogenesis [25,26,69].

## 7. RIPK1 and AD

The kinase RIPK1 is part of the necroptosis pathway and is activated by TNFα, FAS-ligand (FASL), and TNF-related apoptosis-inducing ligand (TRAIL) after contact with their receptors belonging to the death receptor family [70]. Neurons are sensitive to this form of cell death when RIPK1 is associated with RIPK3 and mixed-lineage domain-like protein (MLKL), and in the absence of activated caspases. RIPK1 also induces apoptosis after dimerization and ubiquitination and also mediates inflammatory responses in microglial cells. The phosphorylation of RIPK1 by mitogen-activated protein kinase kinase kinase 7 (TAK1) also seems to play a role in RIPK1-mediated cell death [71]. Under certain conditions, IFN can trigger the association between PKR, RIPK1, and RIPK3 and induce necroptosis [72]. Recent studies have demonstrated that MK2 phosphorylates RIPK1 and prevents TNFα-induced cell death and controls RIPK1 signaling in inflammation [27,69,73]. Necroptosis is observed in AD brains [74,75]. In addition, the RIPK1/RIPK3 complex is activated by LPS and Toll-like receptor 4 (TLR4) and induces inflammation [76]. A link between RIPK1 triggering and inflammation mediated by microglial cells in AD was recently demonstrated [77]. The results showed that in AD transgenic mice, RIPK1 induces a disease-associated microglia pattern with Cystatin F expression. The resulting abnormal lysosomal function could lead to enhanced Aβ 1-42 accumulation and neuroinflammation. Recently, a CNS-penetrant RIPK1 inhibitor (DNL104) was developed and tested in a phase 1 study including 68 healthy volunteers [78]. DNL104 blocks RIPK1 phosphorylation and is generally rather well tolerated, but liver toxicity was observed in 37.5% of cases in the multiple ascending dose groups. Another phase 1b study to evaluate the safety, tolerability, pharmacokinetics, and pharmacodynamics of DNL747, another RIPK1 inhibitor, in 16 AD patients is ongoing in the US and the Netherlands (NCT03757325, https://clinicaltrials.gov, accessed on 1 January 2021).

## 8. Conclusions

### Targeting Several Kinases in AD Patients

The use of combination therapy in humans has not been frequently tested in neurological patients but has been proposed in cancer clinical research in patients with melanoma, for example [79]. Treatment with serine/threonine-protein kinase B-Raf (BRAF) and mitogen-activated protein kinase kinase (MEK) inhibitors results in increased progression-free and overall survival. Aβ neurotoxicity is attenuated in vitro by concurrently blocking PKR and JNK [80]. To our knowledge, combination therapy with kinase inhibitors has not been tested so far in AD patients. Regarding the current signaling pathway PKR/p38/RIPK1, only p38 inhibitors and RIPK1 inhibitors have been explored in controls and AD patients [9,78,81,82]. Pharmacological research is also focusing on PKR and MK2 inhibitors which could also interfere with abnormal brain signals detected in AD brains. PKR and p38 inhibitors could also reduce abnormal tau phosphorylation. Following the findings described in cancer research, it is reasonable to assume that acting early in the evolution of AD brain lesions by using lower doses of combined inhibitors and at several levels of the deleterious signaling cascade might be more efficient to delay neurodegeneration than a single inhibitor treatment. This new pharmacological approach, favored by the possibility to detect early brain lesions with blood biomarkers in cognitively normal individuals and mild cognitive impairment (MCI) patients [83], could be put in place as soon as new kinase inhibitors are tested in humans in order to alter the relentless cognitive decline of AD patients. So far, no disease-modifying treatment has been approved for Alzheimer’s disease by regulatory authorities. Currently, major clinical trials are focusing on immunotherapies aimed at reducing the brain concentrations of Aβ peptides, targeting monomers, oligomers, or plaques, or at decreasing brain concentrations and spreading of phosphorylated tau protein [84]. Other therapeutics such as drugs interfering with neuroprotection or neuroinflammation are also presently being tested in AD or MCI-AD, but clinical studies will be completed in the near future [85].

Regarding the PKR/p38/RIPK1 pathway and AD, the positive finding is that all of these kinases are abnormally activated in AD brains and could also be implicated in the production of amyloid-beta and in tau phosphorylation. They represent valuable therapeutic targets, but unfortunately, so far, clinical trials using a single kinase inhibitor have not been successful [86]. The weakness in this field is linked to the lack of clear information about the onset of abnormal activation of these kinases in the AD brain. This event could occur during the preclinical phase, which can last for one or two decades, or at the MCI stage. Interestingly, CSF levels of PKR are already increased in MCI patients, but further evaluations of CSF levels of other kinases are needed in large cohorts of AD patients. Perhaps in the future, the assessment of blood kinase levels in selected patients will be a new and useful way to implicate the PKR/p38/RIPK1 pathway in early AD clinical features. The ultimate goal in AD therapy is to slow or block neurodegeneration (disease-modifying therapy) and alter the relentless cognitive decline. If these kinases were activated early in AD evolution and correlated with other biomarkers such as Aβ1-42, tau, and phosphorylated tau and cognitive decline, this could be a major incentive to envisage the use of early combination therapy with kinase inhibitors in AD patients. p38 and RIPK1 inhibitors have already been tested in humans, and active research is ongoing concerning PKR inhibitors. From our point of view, the combined inhibition of several kinases belonging to a well-described signaling pathway such as the PKR/p38/RIPK1 pathway could bring about a way to target several key points in the detrimental cascade leading to neurodegeneration, neuroinflammation, and cognitive decline. One of the biggest challenges to solve would be to determine the efficient combined concentrations of inhibitors, to circumvent side effects, and to administrate this treatment as early as possible, knowing the results of biomarker studies. The major interest of this neuroprotective approach is to modulate abnormal signaling when neurons are not too damaged and able to respond to a therapeutic intervention. Cognitive assessments as well as imaging and biomarker evaluations represent the best accurate approach to observe a neuroprotective strategy in treated patients. The time has come to try new therapeutic interventions in AD in order to attenuate the relentless cognitive decline and the increasing burden of affected individuals and their families.

## Figures and Tables

**Table 1:** Non-exhaustive list of articles showing an involvement of the PKR/p38/RIPK1 pathway in patients with Alzheimer’s disease and in animal models.

PKR	Accumulation of Neuronal PKR in AD Brains	Chang et al., 2002
PKR	Increased levels of CSF PKR in AD patients	Mouton Liger et al., 2012
PKR	CSF PKR levels correlate with cognitive decline	Dumurgier et al., 2013
MKK6	Increased stainings of MKK6 in AD brains	Zhu et al., 2001
P38	Increased stainings of P38 in AD brains	Hensley et al., 1998
P38	Increased levels of p38 in AD brains	Zhu et al., 2000
P38	Early activation of p38 in AD brains	Sun et al., 2003
RIPK1	RIPK1 mediates microglial activation in AD	Offengeim et al., 2017

## Data Availability

Not applicable for this review.

## List of Abbreviations

Eukaryotic translation initiation factor 2-alpha kinase 2 (PKR); Dual specificity mitogen-activated protein kinase kinase 6 (MKK6); p38 mitogen-activated protein kinases (p38 MAPK); MAP kinase-activated protein kinase 2 (MK2); Receptor-interacting serine/threonine-protein kinase 1 (RIPK1); Receptor-interacting serine/threonine-protein kinase 3 (RIPK3); Integrated Stress Response (ISR); Eukaryotic translation initiation factor 2-alpha kinase 3 (PERK); Eukaryotic translation initiation factor 2-alpha kinase 1(HRI); Eukaryotic translation initiation factor 2-alpha kinase 4 (GCN2); triggering receptor expressed on myeloid cells 2 (TREM2); Unfolded Protein Response (UPS); c-Jun N-terminal kinases (JNKs); Toll-like receptor 4 (TLR4); mixed-lineage domain-like protein (MLKL); activating transcription factor 2 (ATF2); Mitogen-activated protein kinase kinase kinase 7 (TAK1); serine/threonine-protein kinase B-Raf (BRAF); mild cognitive impairment (mci).
